# The Purpose of the Hospital

**Published:** 1921-11

**Authors:** 


### THE PURPOSE OF THE HOSPITAL.

By The Secretary-Superintendent of the Middlesex Hospital.

So much has been said and written during the past few years with regard to the unsatisfactory financial position of our Voluntary Hospitals that one feels a natural hesitancy in making any further contribution to the literature on the subject. There is, however, one aspect of the case which has undoubtedly been overlooked, or only touched upon so lightly that its true lesson has not been sufficiently taught. An attempt is here made to define once more the real functions of these institutions, and to indicate upon whom responsibility for their maintenance ultimately rests.

The Voluntary Hospitals have before them three intimately connected purposes which, arranged in order of importance, are as follow:

1. To teach the coming generation of medical men, and to inspire in them the desire, not merely to practise the best that is known, but also themselves to take their share in the advancement of knowledge.
2. To carry out research work; for it is only in the laboratories of the hospitals that the necessary steps of investigation can be taken under those exact and perfectly controlled conditions which are essential to the evolution of new discoveries in medical science.
3. To afford treatment to those whose circumstances are such as to debar them from obtaining the highest standard of medical and surgical skill at their own expense.

Blame the war for the present condition of affairs, if you will; deplore the apathy shown by the general public to all questions which relate to the work of those institutions, and insist that all who benefit directly as patients from their activities should contribute according to their means towards the cost of their maintenance, but do not exclude from criticism, or comment, the hospitals themselves. For a fair question to ask is whether the hospitals themselves are not in some measure to blame for the condition in which they now find themselves.

Though your business man will expose in his windows the goods which attract and are popularly sought after, he will not obtrude them upon the notice of the public to the exclusion of the sale of other wares more costly but more lasting, which are stored less conspicuously on his premises, but meet the primary and permanent demands of his customers.

Yet the hospitals by their propaganda unceasingly dwell upon the plight of the suffering poor and the efforts which they make to relieve this special class, thereby creating an impression that this is their sole mission, and that they must rely wholly upon emotional appeals to the charitable for the support they need. No doubt such methods proved more or less adequate when costs were lower than now, when the claims of charity could be met from surpluses, or the charitable instinct be gratified as a mild indulgence to generous feeling; but to-day too many people have to think before they give, others, who could afford to give, thoughtlessly neglect their opportunities, whilst others are still prepared to give and give freely towards objects in which they are interested and of whose value they are convinced. So hospital administrators in general deplore the insufficient yield of the springs of charity as their only source of supply, when charity should be a means ancillary to other and surer channels of support.

How can the public be expected to take genuine interest in the work of hospitals and to support them in their activities unless adequate efforts are made to let them know what are the prime functions of a hospital? Along the path of research there lies within measurable distance the certain goal of further alleviation of human suffering, and, it may be, of the total prevention of many incapacitating or fatal diseases. The triumphs of scientific medicine constitute one of the revelations of the Great War; and there is scarcely a household in the kingdom which has not cause to think with gratitude of benefits conferred on its members by the results of the work carried out in hospital laboratories. "Disease, not battle, digs the soldier's grave" proved a terrible truth in all previous campaigns. The revolution wrought by the application of scientific methods is perfectly summed up in these words, which occurred in one of Lord Haig's despatches: "There has been an almost complete absence of wastage due to disease of a preventable nature."

What capital is to the world of commerce, that, and more, is research to medicine; research is an investment which yields a goodly return in the prevention of pain and the preservation of life and health—inexhaustible benefits which are freely shared by all, and in which each one of us will some day thankfully participate. There is practically no limit to the triumphs that may be achieved in this direction, if research is given proper encouragement and assistance.

It is certain that if the public were given opportunities to see for themselves what the hospitals are doing in this connection, not for the suffering poor alone, but for every individual member of the community, the word "Charity" would soon give place to the words "Obligation" or "Gratitude" as expressing the ground upon which support from them was due. Self-interest too might also be defined, and truly, as a motive which should influence their action.

Take, again, all the classes above those which obtain direct treatment from the hospitals. The skill of the general practitioner or of the consultant depends upon the efficiency of the hospitals and the scientific progress made by them, yet those who praise the cleverness of the doctors who treat them privately rarely realise that without the hospital in their capacity of training-schools, and without the results of the researches carried on in their laboratories, the

**The Purpose of the Hospital—(*cont.*).**

skill now exercised for their benefit would never have been attained.

Unless and until the public grasp the essential truth that the immunity of themselves and of their children from sickness and disease rests wholly with the hospitals, and that, given proper support, the organisation of the hospitals can assuredly reduce still further the incidence of disease which stands a constant menace to their length of life, their happiness and their natural well-being, these institutions will never be in a position to do to the full extent what they are capable of doing, but must prosper or languish as "Charity" dictates.

Let all the hospitals, therefore, teach the same doctrine of principle and purpose, let them encourage the public to see for themselves what they do, apart from the treatment of the sick, then with greater understanding will come the sympathetic interest and practical help which are so urgently needed.

A movement in this direction has already been made at the Middlesex Hospital. Invitations have been issued to employees of firms in the neighbourhood and to others to visit the hospital on set evenings, when the visitors are shown, not the wards, with whose work they are already familiar, but the scientific departments where interesting exhibits are arranged for their inspection, and lectures and demonstrations are given for their enlightenment. The opportunity to gain an insight into the inner working of the hospital's organisation has been welcomed and readily accepted, with the result that a different and truer conception of the hospital's purpose and of its value to the whole community has been formed in the minds of those who previously had no knowledge of the scope of its work, or that it had any purpose beyond that of caring for the sick.

Interest, appreciation, and practical sympathy will be found in every section of society when once the situation is rightly understood, and it should be the task of the hospitals to see that the several services they perform are explained in order of importance, and that in asking for the means to carry on and extend their work they are not pleading only for the poor, but for power to serve and free from suffering every man, woman, and child, and to make for them and those who follow a cleaner and healthier world.

---

There is no greater trap for the unwary than the hot-water bottle which is prone to leak at the neck, or the material of which cannot be depended on to stand the maximum of wear. With the detachable rubber washer there is also the risk of loss or breakage at the psychological moment without another at hand to replace it. The patent washer, therefore, which provides an absolutely water-tight stopper is nothing less than a necessity. This improvement, with a constructed "neck" to ensure the permanent fixing of the necessary brass socket to the rubber in such a manner as to prevent leakages, is incorporated in the "Eclipse" Hot-Water Bottle manufactured by Messrs. Ingram. This firm has been making hot-water bottles since 1847, and its technical experience is constantly employed to secure perfection.

### THE PENSIONS HOSPITALS.

### Bellahouston Inquiry and Report.

Since the Committee appointed by the Minister of Pensions to inquire into the management of Bellahouston Hospital, under the Ministry and the Joint Disablement Committee for South-West Scotland, has presented an unanimous report virtually acquitting the hospital, detailed notice of the findings are superfluous.

The main allegations concerned the out-patient department and the discharge of patients. On December 1, 1919, the hospital was taken over by the Ministry of Pensions. Till the following February the Committee finds no reasonable cause for complaint in the circumstances, but from February 1920 the number of patients multiplied enormously, and there were several grounds for the complaints made, though the Committee of Inquiry is careful to add that the paucity of the staff, and not its zeal, was to blame. On January 18, 1921, Colonel Henchley was appointed medical superintendent, and called the attention of the Joint Disablement Committee to the congested accommodation and small staff in the out-patient department. He tried to reduce the number by weeding, and, after examination, between February 5 and 12, 953, or about half, were discharged.

The Committee of Inquiry says on this, "unless we challenge the capacity or truthfulness of the medical men who conducted this examination and appeared before us, it is impossible in our opinion to discredit the results at which they arrived. We find no sufficient grounds for making such challenge. . . ."

The International Union of Ex-Service Men asserted that a considerable number of those discharged at this time were quickly readmitted as in-patients. But, surveying the readmissions recorded by Colonel Henchley, the Committee thinks that the number of well-founded complaints was very small, and "the department is now in good working order."

In regard to the 300 in-patients discharged in April 1921, the Committee says that these discharges arose out of the industrial crisis. The possibility of a stoppage of transport had to be confronted. The Committee says that the work was carried out sympathetically, that no patients were forced to go home, and almost all were pleased to leave. The allegation that "some patients who had been 'prepared for immediate operation' had been discharged in April 1921" is dismissed by the Committee as supported by "no evidence whatever." "In our opinion," the Committee proceeds, "the allegation is entirely untrue."

At first, the Committee admits, there may have been, and no doubt were, mistakes when the hospital was taken over, and in the new out-patient department, but, generally speaking, the hospital is declared to have served its purpose and done its work well.

We give the above summary of the Committee's findings, not only because the case of all ex-Service men must be considered with sympathy, and, indeed, generosity, but because these pensions hospitals have of late been generally attacked in the lay newspapers.

---

The Committee of the Yarrow Home for Convalescent Children, Broadstairs, is anxious that it should be generally known that the fees payable for admission of children are dependent in a measure on the means of the parents. The basic payment at present is 21s. per week, but this is subject to addition or deduction according to the means of the parents.

